# PTTM: dissecting the profile of tumor tissue microbiome to reveal microbiota features and associations with host transcriptome

**DOI:** 10.1093/bib/bbaf057

**Published:** 2025-02-10

**Authors:** Ruiqian Yao, Lu Sun, Ruifang Gao, Yue Mei, Geng Xue, Dong Yu

**Affiliations:** School of Health Science and Engineering, University of Shanghai for Science and Technology, Shanghai 200093, China; Department of Medical Genetics, Naval Medical University, Xiang-Yin Road, 800, Shanghai 200433, China; Department of Dermatology, Naval Medical Centre, Naval Medical University, Shanghai 200052, China; Department of Precision Medicine, Translational Medicine Research Center, Naval Medical University, Xiang-Yin Road, 800, Shanghai 200433, China; Shanghai Key Laboratory of Cell Engineering, Shanghai, China; Department of Precision Medicine, Translational Medicine Research Center, Naval Medical University, Xiang-Yin Road, 800, Shanghai 200433, China; Shanghai Key Laboratory of Cell Engineering, Shanghai, China; Department of Precision Medicine, Translational Medicine Research Center, Naval Medical University, Xiang-Yin Road, 800, Shanghai 200433, China; Shanghai Key Laboratory of Cell Engineering, Shanghai, China; Department of Medical Genetics, Naval Medical University, Xiang-Yin Road, 800, Shanghai 200433, China; Department of Precision Medicine, Translational Medicine Research Center, Naval Medical University, Xiang-Yin Road, 800, Shanghai 200433, China; Shanghai Key Laboratory of Cell Engineering, Shanghai, China

**Keywords:** pan-cancer, tumor tissue microenvironment, tumor-promoting/suppressing microbes

## Abstract

Microbiota is present in the human tissue microenvironment and closely related to tumorigenesis and treatment. However, the landscape of tissue microbiome and its relationship with tumors remain less understood. In this study, we re-analyzed the omics data from the 7104 samples (94 projects for 15 cancers) in the NCBI database to obtain microbial profiles. After normalization and decontamination processing, we established classification models to distinguish between different tumors and tumor with adjacent normal tissues. The models had excellent performances, indicating that tissue microbiome had significant tumor specificity. Moreover, a series of key bacteria and bacteria-gene association pairs were screened out based on bioinformatic analysis, such as the tumor-promoting bacteria *Fusobacterium*, the tumor-suppressing bacteria *Actinomyces*, and the significant *Rhodopseudomonas*-*COL1A1* association pair. In addition, we created a visual website, PTTM (http://198.46.152.196:7080/), for users to query and download the results. The identified key bacteria and association pairs provide candidate targets for further exploration of the molecular mechanisms of microbial action on tumorigenesis and the development of cancer therapy.

## Introduction

The human body has an estimated 3 trillion bacterial members that coordinate a comprehensive interplay of physiological processes and disease susceptibility [1]. In addition to bacteria, the human microbiome includes eukaryotic fungi and protozoa as well as viruses [2]. These microorganisms are known to colonize the gut, skin, mouth, urine, and a variety of other organs. Growing researches show that organs and tissues previously considered sterile, such as the lungs, prostate, bladder, breast, liver, and pancreas, are now identified to have potentially low-biomass microbial communities [3–6], which are found to play important roles in tumorigenesis and development [7]. According to statistics, 20% of tumors are driven by infection by pathogenic microorganisms, and human commensal microorganisms also influence tumorigenesis and development in terms of immune homeostasis and material metabolism [8]. For example, *Helicobacter pylori* induces the degradation of the cancer-suppressing protein p53 in gastric epithelial cells, leading to gastric cancer [9]. *Fusobacterium nucleatum* can curtail the killing ability of NK cells to tumor cells and thus inhibit antitumor immunity in colorectal, breast and pancreatic cancers [5, 10–12]. The *non-enterotoxigenic Bacteroides fragilis* derived bile salt hydrolase was reported to potentiate colorectal cancer by activating the β-catenin/CCL28 axis to elevate intra-tumoral immunosuppressive CD25^+^FOXP3^+^ T_reg_ cells [13]. In a recent study, Sicheng Wu et al. collected a set of human gut metagenome data derived from healthy and diseased individuals, including cancer patients, to build a curated database of consistently annotated human gut metagenomes to identify marker taxa for different types of cancers, such as *F. nucleatum*, *Parvimonas micra,* and *Gemella morbillorum* in colorectal cancer [14]. These studies confirm that microorganisms are pervasive among tumor tissues, and exploration of the microorganisms involved in tumorigenesis has received a great deal of attention.

Recent studies suggest that microbial presence can be derived from sequencing data (whole genome sequencing [WGS], whole exome sequencing [WES], and RNA) by sequence realignment to the microbial reference genome database. Rodriguez et al. revealed the bacterial composition of nine cancer types using whole exome sequencing data from The Cancer Genome Atlas Program (TCGA) and found significant differences in bacterial shifts between tumor and adjacent normal tissue across stomach, colon, lung squamous cell, and head & neck cohorts [15]. Anders B. Dohalman et al. established a collection of curated, decontaminated microbial compositions of oropharyngeal, esophageal, gastrointestinal, and colorectal tissues based on the reanalysis of TCGA data. In addition to microbial profiling identification, this method also enabled systematic matched microbe-host multi-omics analyses, which will help guide future studies of the microbiome’s role in human health and disease. Anders B. Dohalman discovered a set of prognostic species and blood signatures of mucosal barrier injuries [16]. Kaipu chen et al. provided a database of cancer-associated bacterial information, including the relative abundance of bacteria, associations with clinical relevance, co-expression network of bacteria and human genes, and their associated biological functions [17]. These studies provide us with a new strategy for the study of tissue microbiome. In addition to TCGA, there are many public databases, such as NCBI, EBI, and NGDC, which provide a large amount of tumor multi-omics data to reuse.

Although it is widely accepted that microorganisms are ubiquitous in the tumor tissue microenvironment, the landscape and features of tumor microbiome in different cancers remains unknown. Few studies and databases have focused on microbes colonizing solid tumor tissues, especially the prostate, pancreas, liver, renal and other non-digestive organs. And conventional microbial detection methods (including 16S amplicon sequencing and metagenomic sequencing) are not effective in detecting microbial information in tumor microenvironment due to the low mass compared to tissue cells. However, the existing public databases provide us with large amount of open-accessed omics data for exploring the tissue microbiome in tumor environments. With this in mind, we integrated and analyzed the sequencing data from 7104 samples of 15 cancer types in the NCBI Sequence Read Archive (SRA) database, including RNA sequencing, WGS, and WES data, to establish the profile of tumor microbiome. Based on the profile, we established tumor classification models and screened a series of cancer-promoting/cancer-suppressing microbes, hub microbes that play key roles in the co-occurrence network, and genus-gene significant association pairs. These key microbes are the candidate targets to contribute to tumorigenesis, tumor diagnosis and treatment, and the development of related drugs. All the results are visualized on the website, and the data generated in this study are uploaded to the website for public download and reuse (http://198.46.152.196:7080/).

## Materials and methods

### Data collection and preprocessing

The study included 15 types of cancer: bladder cancer, brain cancer, breast cancer, cervical cancer, colorectal cancer, endometrial cancer, esophagus cancer, gastric cancer, liver cancer, lung cancer, lymphoma, ovarian cancer, pancreatic cancer, prostate cancer, and thyroid cancer. We searched the projects that met the criteria in the NCBI SRA database and summarized the ProjectID, RunID, and phenotype of the samples. The selection criteria were as follows: (i) tumor or adjacent normal tissues of solid tumors of human origin; (ii) the data came from the sequencing data of RNA-seq, WGS and WES; and (iii) the number of samples in the project was greater than 15. The phenotype information was finally summarized into one biological (source: tumor versus adjacent normal tissue) and three technological (Assay type, Center name, Instrument) factors. Detail information of these samples was present in supplementary Table 1.

The raw data from all projects were downloaded in the SRA format and converted into FASTQ format using the SRA Toolkit (version 3.0.1). The raw FASTQ files were processed for quality control using FastQC (version 0.11.9) and Trim Galore (version 0.6.7) software. The ultrafast Karen2 algorithm was used to identify the microorganisms. The microbial reference database contains 83,212 genomes, which include almost all known fungal, bacterial, archaeal, and viral genomes. Moreover, RNA-seq data were aligned with the human genome (GRCh38) using HISAT2 (version 2.2.1), and the gene expression profiles were then obtained using Stringtie (version 2.2.1).

### Decontamination

A growing number of studies are attempting to conduct decontamination analysis of microbiome data based on different methods in microbiome studies, and many valuable contaminant ‘blacklists’ have been generated. We referred to Poore et al. from four levels to determine the ‘blacklists’. Their data are available in the online repository (ftp://ftp.microbio.me/pub/cancer_microbiome_analysis). This produced four filtered data in our study: (i) filtered data1: likely contaminants removed by the sequencing center (be short for Likely); (ii) filtered data2: all putative contaminants removed by the sequencing center (be short for Putative); (iii) filtered data3: most stringent filtering by the sequencing center (be short for Most); and (iv) filtered data4: contaminants removed by sequencing plate center combinations (be short for Plate).

As a further conservative measure, we created the fifth filtered data based on *mbodymap*, a curated database of microbes from 22 body parts in healthy and diseased individuals [18]. On the website (https://mbodymap.microbiome.cloud), we screened the genus taxid and obtained 1338 genera as a ‘whitelist’. Based on its criteria, we re-examined the samples of each project to ensure that the species included in the analysis in each project were present in two or more samples and finally overlapped with 1338 genera to obtain the filtered data5: Overlapped with mbodymap (be short for Ombodymap).

### Normalization

In big data processing and analysis where multiple projects are merged, differences in sequencing technologies, sequencing instruments, sequencing centers and other technical factors can confound the results of data analysis. Therefore, we need to eliminate the effects of these batch effects while retaining the biological information that the data have.

We employed a pipeline that was Voom transformation and supervised normalization (SNM) to normalize the data [19, 20]. Briefly, we first performed the Voom transformation to remove the anisotropy of the data. For most of the datasets (raw data, ‘Likely’, ‘Putative’, ‘Plate’, ‘Ombodymap’), normalization was done using the weighted trimmed mean of M-values (TMM), while removing reads that were invariant across multiple groups. For the data ‘Most’, quantile normalization was used because these data already had significantly reduced microbial features and read counts. The Voom transformation data were then applied in the SNM model, where we used the known expected biological differences between sample types (tumor versus adjacent normal tissue) as target biological variables. The following technical variables were taken as covariates to be mitigated during SNM: Center name (sequencing center, n = 78), Instrument (sequencing instrument, n = 8), and Assay type (sequencing technology, n = 3). For the host gene expression profile, the normalization process was the same as above.

To estimate the impact weights of these factor variables before and after normalization, we quantified the changes between the raw, Voom transformation, and Voom-SNM normalized data using principal variance analysis (PVCA, https://www.niehs.nih.gov/research/resources/software/biostatistics/pvca/index.cfm) [21, 22].

### Classification model

The Gradient Boosting Machine (GBM) algorithm is a kind of Boosting algorithm, which has high prediction accuracy and can effectively fit complex nonlinear relations. We used the R-package gbm (version 2.2.2) model to establish two types of classification models in this study: (i) among tumors: distinguishing one cancer type from all other cancers; (ii) within tumors: distinguishing tumor from adjacent normal tissues of one cancer type. The training and test datasets were conducted in 70% and 30% of the total data respectively, randomly selected, stratified sampled, and seeded with fixed random numbers to ensure repeatability of model results and comparability between models. Then the Receiver Operating Characteristic (ROC) curve is generated and the Area Under the Curve is calculated to measure the performance of the model. GBM models can estimate the importance of each feature in the model, helping to understand the data and make feature selection. R-package Caret (version 6.0–94) was used to calculate the importance scores of variables (i.e., genera) in the model. The higher the scores, the more important the genera are to distinguish the model.

### Statistical analysis

All statistical analyses were conducted in R (version 4.2.3). The microbial profiles were transferred into the phyloseq format using the package phyloseq (version 1.42.0). The related microbiome analysis, including diversity analysis, differential analysis, network analysis and association analysis, was performed using the package microbiome (version 1.20.0), vegan (version 2.6–4), and psych (version 2.3.6).

In the diversity analysis, the Shannon index was calculated, and the *P* of the Wilcoxon test less than 0.05 was considered significant at the group level. The package Maaslin2 (version 1.12.0) was employed to detect the differentially abundant microbes between the tumor and adjacent normal tissues with the threshold (*P* < .05). The co-occurrence network of genera was analyzed by the Meconetcomp R package [23], with the threshold of Spearman values (*P* < .05 & r > 0.8). The network topology is visualized by Gephi software, node color represents phylum, node size represents degree, edge red represents positive correlation and blue represents negative correlation. For the topological features of genera, we calculated the values of the within-module connectivity (Zi) and among-module connectivity (Pi) to divide genera into four groups to reveal their ecological roles. Apart from Peripherals, the remaining three types of nodes (Module hubs, Connectors and Network hubs) are usually classified as critical nodes since these nodes are in the hub position [24]. Differentially expressed genes were detected using the R package ‘limma’, with a cutoff of 2 for log-transformed fold change and *P* < .05. The Kyoto Encyclopedia of Genes and Genomes (KEGG) pathway enrichment of differentially expressed genes was performed using the clusterProfiler R package (v3.16.1).

Spearman correlation analysis was also performed to calculate the correlations between significant differential genera and genes, and the results of the top 10 significantly differential genera and genes are shown in the heatmap.

## Results

### Data processing and normalization

**Figure 1:**
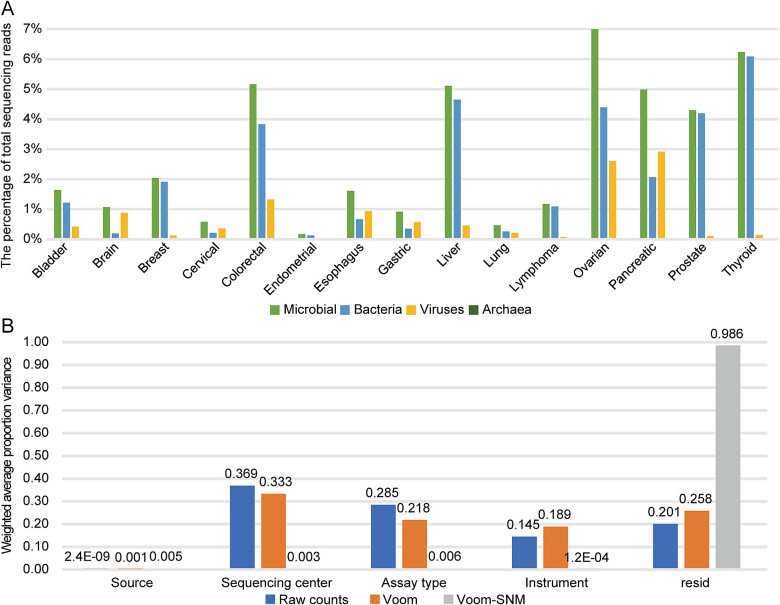
**The distribution of microbial reads and its influence factors.** (A) the percentage of microorganisms, bacteria, archaea and virus in the total sequencing reads. (B) the PVCA of different factors in the microbial distribution.

In this study, we selected 15 types of solid tumors as research objects, which are labeled the top 10 cancers with high incidence and death in China in 2022 [25]. We collected a total of 7104 samples from 94 projects in the NCBI SRA database, of which 77.94% were tumor tissue samples and the rest were adjacent normal tissue samples. The details are shown in Table 1. Through our process, 2.59% of 5.31 × 10^7^ sequencing reads of an average per sample were classified as microorganisms (bacteria, viruses, and archaea) identified by kraken2, of which 73.78% (1.91% of total reads) were bacteria (Fig. 1A). Considering the dominant role of bacterial taxa, the following analysis focused on bacteria. The microbial taxa with fewer than three reads were regarded as false positives and then discarded. Finally, we found a total of 5372 species belonging to 1324 genera, and the following analysis will be performed at the genus level.

**Table 1:** Summary of sample phenotypic information for each cancer.

Cancer type	Projects	Samples	Sequencing center	Source		Assay type		
				tumor	adjacent	RNA-seq	WXS	WGS
Bladder	7	478	7	417	61	478	/	/
Brain	5	209	4	197	12	143	64	2
Breast	8	539	8	494	45	334	205	/
Cervical	4	185	4	157	28	151	34	/
Colorectal	10	1044	10	666	378	553	491	/
Endometrial	2	40	2	31	9	18	22	/
Esophagus	6	454	6	268	186	126	314	14
Gastric	4	726	3	379	347	726	/	/
Liver	9	616	9	393	223	465	102	49
Lung	6	339	5	250	89	204	111	24
Lymphoma	10	519	9	466	53	247	272	/
Ovarian	7	477	6	477	/	379	98	/
Pancreatic	4	446	4	436	10	446	/	/
Prostate	8	484	8	457	27	190	194	100
Thyroid	4	548	4	449	99	187	361	/
Total	94	7104	78	5537	1567	4647	2268	189
**Cancer type**	**Instrument**							
	**Illumina_HiSeq**	**Illumina_MiSeq**	**HiSeq_X_Ten**	**NextSeq**	**Illumina_GA**	**Illumina_NovaSeq**	**BGISEQ**	**Ion_Torrent_Proton**
Bladder	203	8	65	126	76	/	/	/
Brain	88	/	2	/	/	119	/	/
Breast	489	20	/	/	30	/	/	/
Cervical	123	/	28	34	/	/	/	/
Colorectal	89	/	38	126	/	791	/	/
Endometrial	40	/	/	/	/	/	/	/
Esophagus	374	80	/	/	/	/	/	/
Gastric	696	/	/	/	/	/	30	/
Liver	387	19	70	/	/	140	/	/
Lung	201	/	/	5	/	133	/	/
Lymphoma	118	115	41	14	/	228	/	3
Ovarian	232	/	/	195	/	50	/	/
Pancreatic	290	/	62	94	/	/	/	/
Prostate	420	/	/	/	6	58	/	/
Thyroid	548	/	/	/	/	/	/	/
Total	4298	242	306	594	112	1519	30	3

To eliminate batch effects due to the complexity of the collected datasets, we applied a Voom-SNM pipeline (see Methods). The PVCA analysis showed that the Voom-SNM normalized data retained and increased the contributions of biological factors (Source: tumor versus adjacent normal tissue) while effectively reducing the effects of technical factors (Center name, Assay type, Instrument) in each project (Fig. 1B, Fig. S1).

### Contaminant removal

**Figure 2:**
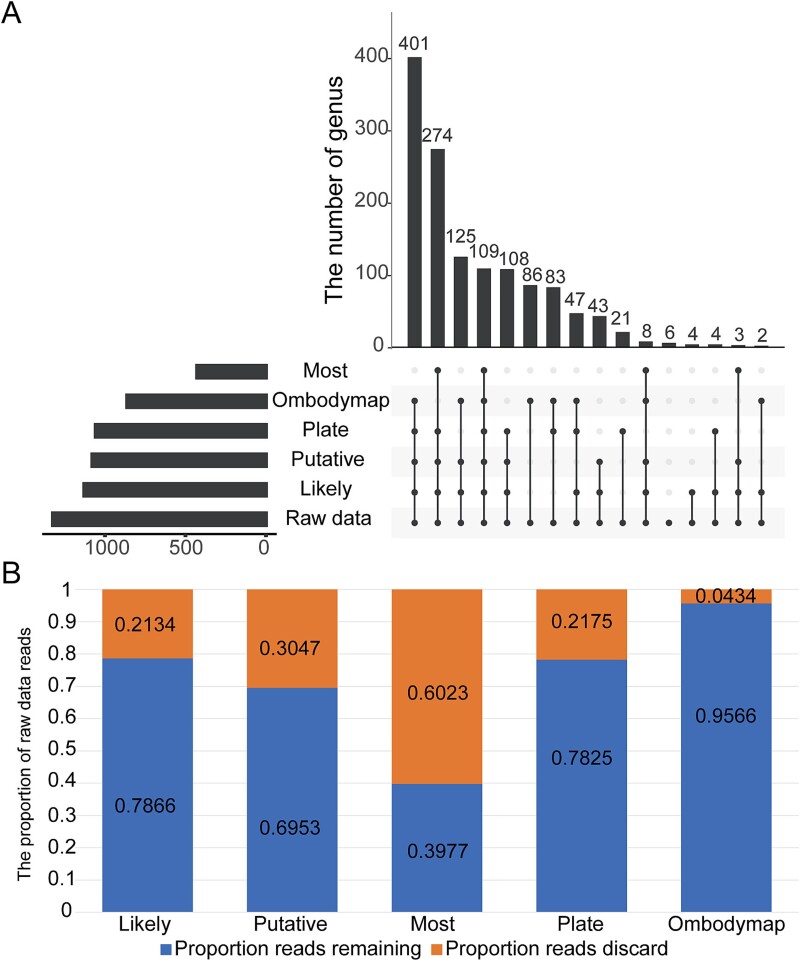
**Distribution of genus and reads in the process of contamination removal.** (A) the number of overlaps of genera in each filtered data. (B) the proportion of remaining and discarded reads after varying levels of filtering. Likely, likely contaminants removed by the sequencing center; putative, all putative contaminants removed by the sequencing center; most, most stringent filtering by the sequencing center; plate, contaminants removed by sequencing plate_center combinations; Ombodymap, overlapped with mbodymap.

To better characterize the microbes associated with cancer, we recognize the importance of mitigating the potential effects of contamination. We created five filtered data points from the perspective of removing ‘blacklist’ contaminants and retaining ‘whitelist’ (see Methods). The five filtered data had a total of 1128 (Likely), 1071 (Putative), 1047 (Plate), 394 (Most), and 861 (Ombodymap) genera. A total of 109 genera were preserved by all filtered data (Fig. 2A). The Most removed 60% of the microbial reads, while the Ombodymap retained 96% of microbial reads despite removing 35% of genera, suggesting that the Ombodymap data may be better at filtering out low-abundance genera (Fig. 2B). Therefore, this study will be based on filtered data5: ‘Ombodymap’ for further exploration.

### Different microbial taxonomic compositions in pan-cancer

**Figure 3:**
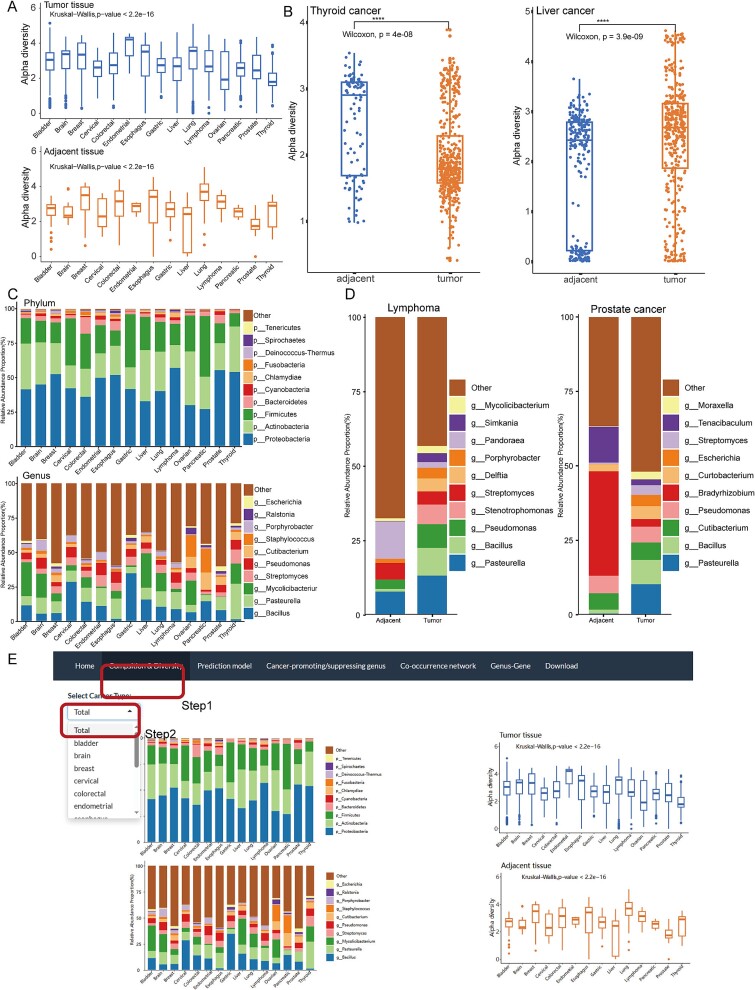
**Microbial diversity and composition among 15 cancer types.** (A) Alpha diversity plot of tumor tissue and adjacent normal tissue microbiome among the 15 cancer types. (B) the alpha diversity plot between tumor and adjacent normal tissues in thyroid cancer and liver cancer. (C) Histogram of the relative abundance distribution of the top 10 phyla and genera among the 15 cancer types. (D) the top 10 genera in the tumor and adjacent normal tissues in lymphoma and prostate cancer. (E) ‘Composition & Diversity’ interface of the PTTM website.

As expected, alpha diversity revealed significant differences among cancer types (Kruskal−Wallis, p − value <2.2e−16, Fig. 3A). The results showed that some gastrointestinal tumors (colorectal cancer, esophageal cancer, gastric cancer) and lung cancer were relatively rich in bacteria in the tissue microenvironment, while cervical cancer had a lower diversity index. We found a higher percentage of reads for viruses in cervical cancer, possibly because the tumor was mainly affected by viruses (Fig. 1A). In addition, some cancers had significant differences in microbial diversity between tumor and adjacent normal tissues (Wilcoxon, *P* < .001, Fig. 3B). For example, adjacent normal tissue microbiota was more abundant in thyroid cancer, which is consistent with previous studies [26]. In contrast, in liver cancer, the alpha diversity in tumor tissues was significantly higher than that in adjacent normal tissues [27].

*Proteobacteria*, *Actinobacteria* and *Firmicutes* were the main phyla that composed the cancer microbiome, and *Bacillus*, *Pasteurella* and *Mycolicibacterium* were the top 3 genera in the 15 types of cancer (Fig. 3C). At the genus level, bacterial profile shifts were observed in tumor tissue compared to adjacent normal tissue in some cancers (Fig. 3D). For example, *Stenotrophomonas*, one of the top 10 genera for lymphoma, had an average relative abundance of 0.07 in tumor tissues compared to 0.0004 in adjacent normal tissues. One of the species belonging to this genus, *Stenotrophomonas maltophilia*, was recognized as a cause of nosocomial infection among immunocompromised individuals [28] and was associated with primary cutaneous anaplastic large-cell lymphoma [29]. In prostate cancer, *Bradyrhizobium* accounted for 35% of the adjacent normal tissue, much more than its abundance in the tumor tissue (2.5%). The antibacterial action of *Bradyrhizobium* has been confirmed in a recent study [30], and it may be a tumor-suppressing microbe in prostate cancer [31]. Therefore, understanding changes in the bacterial profiles of tumors and adjacent normal tissues can provide some insights into the mechanisms of tumorigenesis of microbiota. The details of each cancer can be found on the PTTM website (Fig. 3E).

### Bacteria can be used to distinguish among and within types of cancers

**Figure 4:**
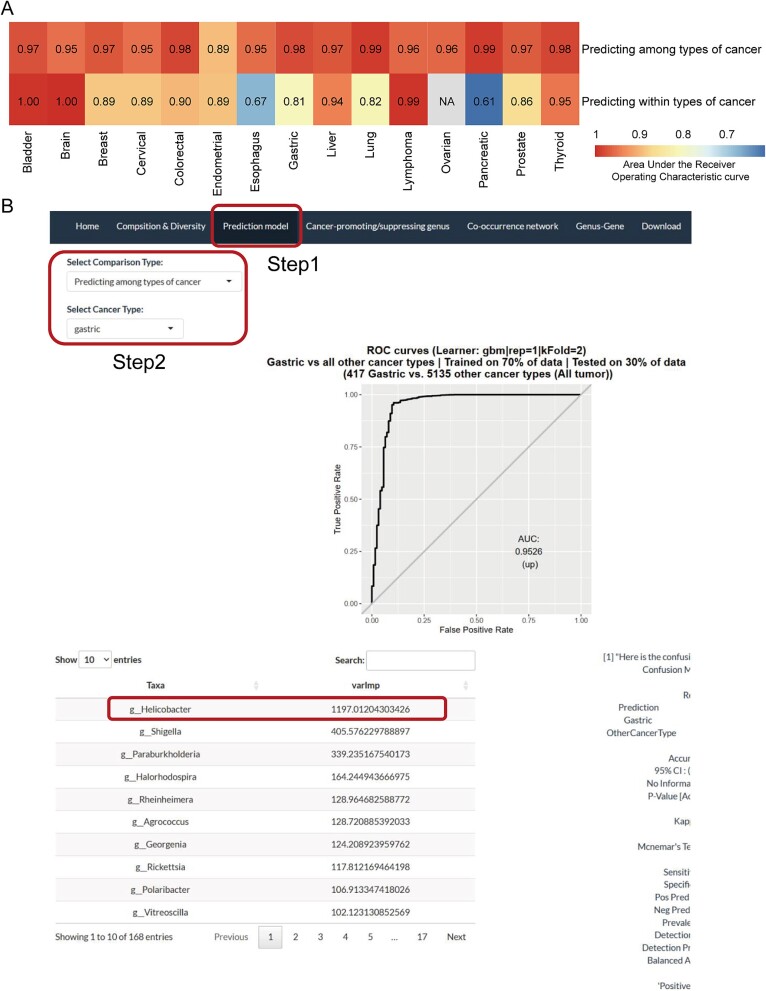
**Performance of ML models to distinguish among and within types of cancers.** (A) Heatmap of AUROC for the models to distinguish among and within types of cancer in Ombodymap. (B) Example of a gastric cancer model in the ‘prediction model’ interface on the PTTM website.

Using normalized data, we constructed machine learning models using stochastic GBM learning models to distinguish among (n = 15 cancers) and within (n = 14 cancers) types of cancer. The models were found to have very good performances, with an average area under the receiver operating characteristic curve (AUROC) of 0.96 among cancers and 0.87 within cancers (Fig. 4A). Together, these results show that the tumor had a type-specific microbiome and significant differences between the tumor and adjacent normal tissues. Similar results were observed with good performances when using the other decontamination categories, which further confirms the specificity of the tumor microbiome and reliability of the models (Fig. S2).

We selected the top 5 genera of each model according to the importance scores and found that some genera were equally important to the multiple models (Table 2). For example, *Bacteroides*, *Faecalibacterium*, and *Streptococcus* commonly found in the gastrointestinal tract appear to distinguish among types of cancer models, and they have been found to be associated with a variety of cancers [32–34]. Moreover, *Psychrobacillus* was effective in distinguishing between tumor and adjacent normal tissues in bladder cancer, colorectal cancer, and liver cancer. Interestingly, in addition to these recurring genera, we found some genera with the highest feature importance to be biologically significant. For example, the well-known carcinogenic microorganism *Helicobacter* is the genus with the highest feature importance to distinguish within gastric cancer; so does *Prevotella* for distinguishing within ovarian cancer [35, 36] and *Shigella* for distinguishing within breast cancer [37]. This suggests that these models had biological implications and were biologically relevant. Detailed information on the models and feature importance of genera are shown on the website (Fig. 4B).

**Table 2:** The top 5 genera in terms of variable importance scores in multiple models.

Genus	Model type	Cancer type	Variable Importance
g__Bacteroides	Predicting among types of cancer	Colorectal	175.44
		Endometrial	620.69
		Prostate	212.19
	Predicting within types of cancer	Bladder	61.44
		Thyroid	43.82
g__Faecalibacterium	Predicting among types of cancer	Cervical	278.53
		Colorectal	323.73
g__Lautropia	Predicting among types of cancer	Liver	268.33
		Ovarian	164.16
g__Prevotella	Predicting among types of cancer	Ovarian	468.80
		Thyroid	150.60
g__Ralstonia	Predicting among types of cancer	Pancreatic	409.61
		Thyroid	163.56
g__Spirochaeta	Predicting among types of cancer	Lymphoma	139.57
		Pancreatic	216.57
g__Tenacibaculum	Predicting among types of cancer	Brain	199.30
		Breast	513.14
g__Herbaspirillum	Predicting within types of cancer	Colorectal	102.48
		Lung	24.10
g__Hymenobacter	Predicting within types of cancer	Colorectal	20.27
		Gastric	18.86
g__Mesorhizobium	Predicting within types of cancer	Gastric	21.12
		Liver	24.19
g__Psychrobacillus	Predicting within types of cancer	Bladder	25.96
		Colorectal	58.50
		Liver	39.57
g__Rhodopseudomonas	Predicting within types of cancer	Esophagus	33.63
		Gastric	16.63
g__Runella	Predicting within types of cancer	Lung	21.13
		Prostate	49.51
g__Streptococcus	Predicting within types of cancer	Breast	23.55
		Lung	14.98
g__Thioalkalivibrio	Predicting within types of cancer	Colorectal	40.47

### Cancer-promoting/suppressing bacteria

**Figure 5:**
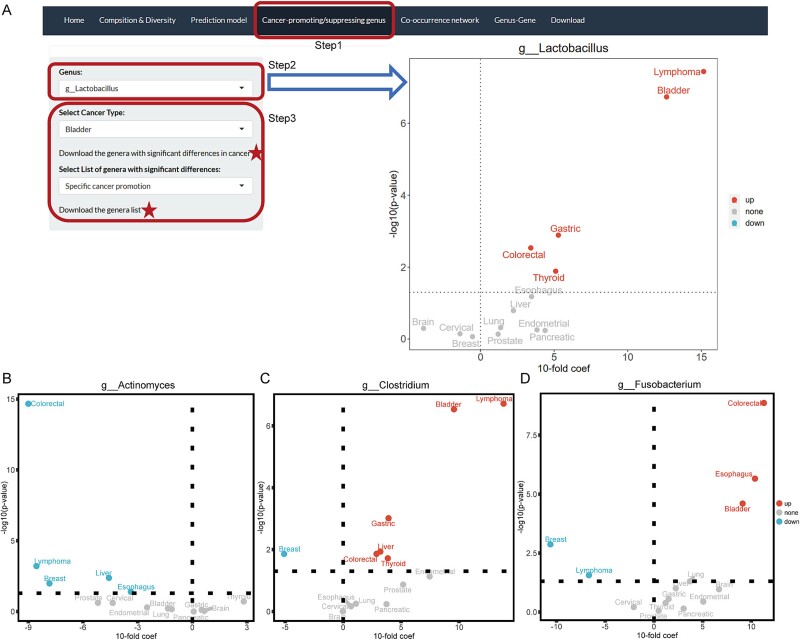
**Examples of cancer-promoting/suppressing genes among the 15 types of cancer.** (A) Example of *lactobacillus* in the ‘cancer-promoting/suppressing genus’ interface on the PTTM website. (B-D) the trend of differential expression of *Actinomyces*, *clostridium*, and *Fusobacterium* in 14 types of cancer. A horizontal dashed line representing the threshold for the p-value and a vertical dashed line representing the threshold for the coefficient. Points in the upper right quadrant indicate significant enrichment in tumor tissue. Points in the upper left quadrant indicate significant enrichment in adjacent normal tissue. Points below the horizontal dashed line indicate no significant difference between tumor and adjacent normal tissue.

To screen cancer-promoting or cancer-suppressing genera, we used normalized data to identify genera with significant differences between tumor and adjacent tissue by MaAslin2 (see Methods). We presented the results of the difference analysis for each cancer on the website and summarized the five lists of (i) promoted (24 genera) and (ii) suppressed (260 genera) in multiple cancers, (iii) promoted (12 genera) and (iv) suppressed (34 genera) in specific cancers, (v) significantly different in multiple cancers (363 genera) for users to download (Fig. 5A).

In the five lists, we found that *Lactobacillus* is significantly enriched in five cancers (bladder cancer, colorectal cancer, gastric cancer, lymphoma, and thyroid cancer, Fig. 5A), and it has been reported to induce anticancer action by enhancing cancer cell apoptosis and protecting against oxidative stress [38]. *Actinomyces* was significantly enriched in adjacent normal tissue in the five cancers (breast cancer, colorectal cancer, esophagus cancer, liver cancer, and lymphoma, Fig. 5B). Several secondary metabolites of *Actinomyces* were reported to have antitumor properties [39–41]. In addition, there are some genera with the distribution of different trends in multiple cancers (↑ indicates a significant increase, and ↓ indicates a significant decrease, in relative abundance in cancer tissue compared to adjacent tissues). For example, we found that *Clostridium* was significantly dysregulated in seven cancers, which was consistent with previous studies (bladder cancer↑ [42], breast cancer↓ [43], colorectal cancer↑ [44], gastric cancer↑ [45], liver cancer↑ [46], thyroid cancer↑ [47], and lymphoma↑ [48], Fig. 5C). *Clostridium* is classified as an oncolytic bacteria, a classification of bacteria that has the natural ability to specifically target solid tumors [49], and infection with *Clostridium* or extracellular toxins has been reported to be associated with tumorigenesis. *Fusobacterium* has been suggested as a potential microbial carcinogen that initiates the development of colorectal cancer [50, 51], and there are currently increasing evidences that the oral pathobiont *F. nucleatum* is involved in the progression of a growing number of cancers. In our results, the expression of *Fusobacterium* was significantly dysregulated in five cancers (bladder cancer↑ [52], breast cancer↓ [5], colorectal cancer↑ [53], esophagus cancer↑ [54], and lymphoma↓ [55], Fig. 5D), which is also consistent with previous studies. In summary, 693 significantly different genera were identified in this study, and our compiled list of cancer-promoting/suppressing genera is expected to provide clues for extensive researchers.

### Hub bacteria in the co-occurrence network

**Figure 6:**
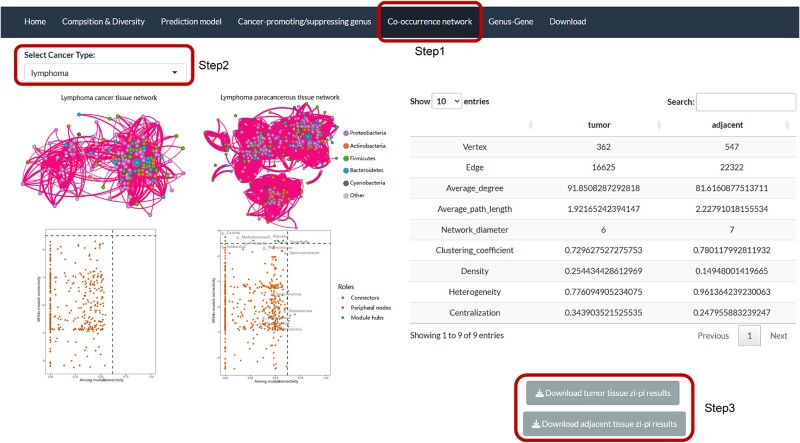
Example of lymphoma in the ‘Co-occurrence network’ interface on the PTTM website.

To understand the potential interactions between genera, we constructed microbial co-occurrence networks of tumor and adjacent normal tissue microbiomes in each cancer using Spearman correlation analysis. The network attributes of each cancer and the topological features of the genera have been shown on the web page for users to download and view (Fig. 6).

In our results, some genera were determined to be hub genera of networks (see Methods), and 68 hub genera played important roles in multiple networks (Table S2). For example, *Synechococcus* is not only a connector of the normal tissue network in lymphoma but also the module hub of the normal tissue network in prostate cancer. *Synechococcus* is classified as a genus of Marine cyanobacteria, which could produce interesting bioactive compounds, particularly for the treatment of cancer [56, 57]. Current studies have confirmed the antiviral/antibacterial activity of methanol extracts from *Synechococcus* on lymphoma and prostate cancer cell lines [58, 59]. A lot of connectors were identified in the tumor tissue networks of cervical cancer, and the network clustering coefficient (0.97) indicates strong bacterial interactions in the microenvironment of cervical cancer tissue. These critical nodes play an important role in maintaining the stability of the network structure.

### The associations of bacteria and host genes

Bacteria colonize human tumor tissue, while the host will devote a particular set of genes to dealing with all potential threats, as well as coordinating benefits with the tissue microbiome. To explore the correlation between human host genes and microbiota, we generated gene expression matrixes from RNA-seq data in this study and identified differential genes between tumor and adjacent normal tissue (see Methods). The website displays KEGG pathway enrichment analyses for each cancer significant differential genes, and the correlation matrixes for significant differential genes and genera are available for download. Overall, 126 and 176 KEGG pathways were significantly enriched in up-regulated and down-regulated differential genes, respectively. Motor proteins appear most frequently in the pathway that up-regulated differential genes. Motor proteins play a critical role in cancer progression by regulating processes such as cell division, migration, and intracellular transport, making them potential therapeutic targets [60]. Among the pathways that down-regulated differential genes, the one that appears most frequently is Neuroactive ligand-receptor interaction. This pathway includes chemokine receptors such as CXCR4 and CXCR7, which are G-protein-coupled receptors activated by their common ligand CXCL12. These receptors are crucial in multiple human cancers, and their antagonism can hinder metastasis by disrupting tumor cell growth, migration, and chemotaxis, while also enhancing T cell infiltration in the tumor microenvironment [61].

**Figure 7:**
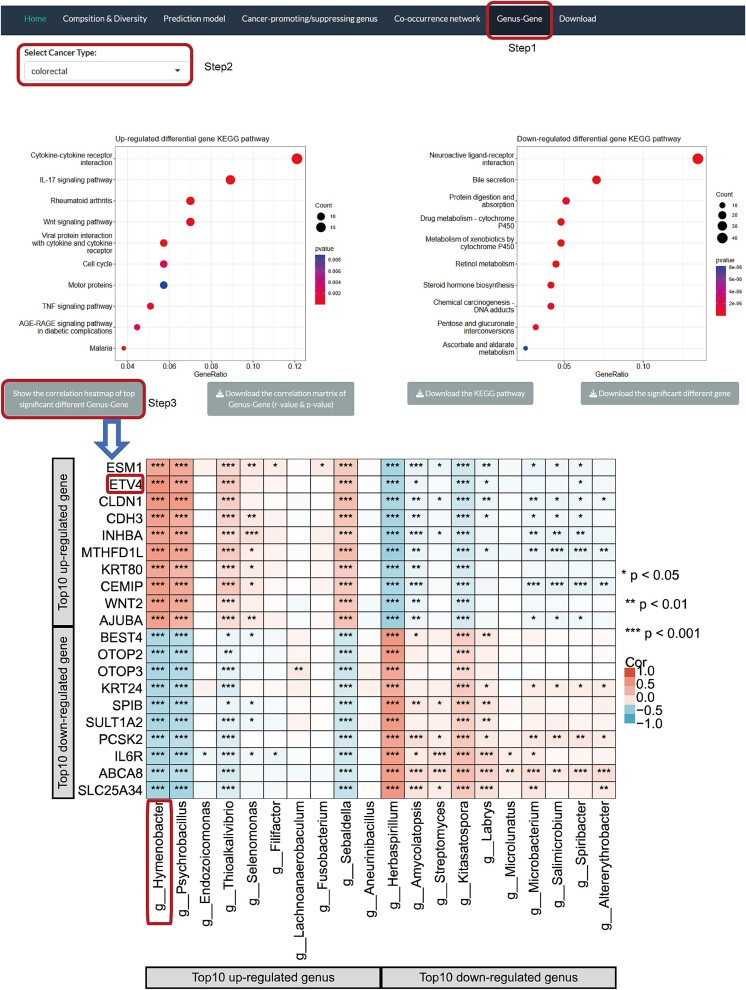
Example of colorectal cancer in the ‘genus-gene’ interface on the PTTM website.

In addition, we generated heatmaps of the correlation between the top 10 differential genes and the differential genera (Fig. 7). For example, in colorectal cancer, we found the significant association pair *Hymenobacter*-*ETV4*. *Hymenobacter* is the most significant cancer-promoting genus, and its abundance was reported to increase significantly in the high-bile acid metabolite group [62]. The high bile acid load is a promoter of colorectal cancer, which indirectly indicates that *Hymenobacter* may be associated with colorectal cancer [63]. As expected, *ETV4*, which is significantly positively associated with *Hymenobacter*, has been identified as a biomarker for colorectal cancer by a previous study [64].

We further focused on 2793 significant association pairs (*P* < .05 and r > 0.5, Table S3) and found that the top five association pairs were present in esophageal cancer (*g__Rhodopseudomonas*- *COL3A1*/*POSTN*/*COL1A2*/*CDH11*/*COL1A1*), while *Rhodopseudomonas*-*COL1A1* was also a significant association pair in colorectal cancer. A previous study found that *Rhodopseudomonas* has tumor-targeting abilities and excellent heat and ROS production, resulting in drastic tumor elimination [65]. The key role of *COL1A1* in esophageal cancer and colorectal cancer has been confirmed by studies [66, 67]. These results indirectly confirm the reliability of our results, and researchers can download a matrix of relevant results from the website to dissect the correlations between the key genera and genes in cancers of interest, providing some hints for subsequent mechanistic exploration.

## Discussion

Microbial communities are abundant in the human body, and microbial dysbiosis can lead to many diseases, including various cancers. Most of the recent researches have focused on the gut microbiome, while the microbes colonized in the tumor microenvironment lack relevant researches. Previous studies have suggested that the presence of microorganisms in tumors and adjacent tissues can inform disease progression and its role in cancer pathogenesis [15]. Therefore, in this study, through the multi-omics data of 7104 samples from 94 projects in the NCBI SRA database, we deeply mined the microbiota of tumor and adjacent normal tissues in 15 types of cancer, screened out some potential cancer-promoting and cancer-suppressing microorganisms, and built a visual website, providing results and raw data for users to query, browse and download.

Compared to previous cancer microbiome databases, our database has several strengths. First, it is the first database to use fully publicly available multi-omics data to explore the microbiome atlas within the tumor tissue microenvironment. Anders B. Dohlman et al. by mining WGS and WES data of gastrointestinal tumors in TCGA, found that *Fusobacterium* and *Bacteroides* were specifically correlated in colorectal tissue microbiota [16]. Bassel et al. developed single-cell analysis of host-microbiome interactions based on public data and found that the most common bacterium associated with cell infection in the tumor cohort was *Campylobacter* [68]. Therefore, public data mining is an effective research strategy that not only has scientific significance but also has significant economic value. Second, a variety of methods were used to minimize batch effects and the impact of contaminants on the results. In microbiological research, the presence of batch effects and pollutants has always posed challenges, particularly when mining public data. This issue becomes more prominent due to factors such as population heterogeneity and technical platform differences. To address these concerns, we employed two distinct methods. One is that we used the Voom-SNM model to reduce the impact of batch effects in the raw data while increasing biological factors. The other is that we carried out five levels of decontamination treatment from the negative and positive perspectives. The filtered data obtained not only retained more of the raw reads (96%) but also performed well on machine learning models (average AUROC 0.96 among cancers and 0.87 within cancers). Third, it is the first batch and systematic screening of a series of key microorganisms in tumorigenesis and development. Some of these microbes, such as *Lactobacillus* and *Fusobacterium*, have been shown in previous experimental studies [68, 69]. In addition, *Actinomyces* and *Clostridium* are consistent with previous data studies [39, 48]. These results demonstrate the reliability of the results and provide potential targets for follow-up studies. Finally, we also established a series of associations between microbiota and host genes. At present, the specific mechanism and path of human microorganisms’ role in tumor occurrence and development are still unclear, and only a few studies have achieved some results through experimental exploration, such as the CagA protein secreted by *H. pylori*, which can also interact with E-cadherin of gastric epithelial cells to promote rapid cell proliferation and increase the risk of epithelial cancer [9]. Compared with the complex microbial composition and gene regulation network, these understandings are still limited, and there are differences among different tumors. The association pairs provided in this study, especially the association between differential bacterial genera and differential genes, will provide potential candidate targets for mechanistic exploration.

Nonetheless, this study has several flaws. First, the relatively small sample sizes for some cancers, such as endometrial cancer, had only 40 samples. Second, the size of adjacent normal samples is relatively inadequate and imbalanced. Only 10 adjacent normal tissue samples were included in the pancreatic cancer. However, we tried different methods to reduce the influence of these flaws on the results. For example, the Voom-SNM normalized pipeline had increased biological signals (tissue source: tumor and adjacent normal) and was chosen to retain microbes from the known human microbiome database *mbodymap* when filtering the data. Next, we will continue to collect and collate public tumor multi-omics data to further improve the microbial profiles of tumor tissue microenvironments. Finally, regarding the key microorganisms selected above, we are also carrying out relevant experiments to explore the molecular mechanisms and provide a reference for peers who carry out relevant research.

## Conclusion

In this study, we revealed the microbial map in the microenvironment of tumor tissue through the mining of massive public databases, analyzed its characteristics systematically and comprehensively, and found a series of key microorganisms, which provided data support and reference for subsequent mechanistic exploration and applied research. All the data and results generated in this study have been stored and visualized in database form on the website, which is open to peers.

**Key Points**

- Present the landscape of tumor microbiome in 15 types of solid tumors based on public omics data mining, which expands our understanding of tumor microbiome;
- Identify a series of potential pro-cancer and anti-cancer microorganisms, providing candidate targets for subsequent mechanism exploration and clinical intervention or therapy researches;
- Identify a series of microbe-gene associations with significance, providing targets for understanding the molecular mechanisms of microorganisms in tumorigenesis;
- Provide an online database to present and share the above results.

## Supplementary Material

FigureS1_bbaf057

FigureS2_bbaf057

Table_S1_bbaf057

Table_S2_bbaf057

Table_S3_bbaf057

## Data Availability

The raw SRA format is available at the NCBI SRA database (https://www.ncbi.nlm.nih.gov/sra/), and the projectIDs and all other relevant data are available at the download page of the website (http://198.46.152.196:7080/). The R source code is available in a supplementary file.
