# Supplementary figures and images for: PTTM: dissecting the profile of tumor tissue microbiome to reveal microbiota features and associations with host transcriptome
Source: Brief Bioinform. 2025 Feb 10;26(1):bbaf057. doi: 10.1093/bib/bbaf057 (PMC11807729; doi:10.1093/bib/bbaf057)

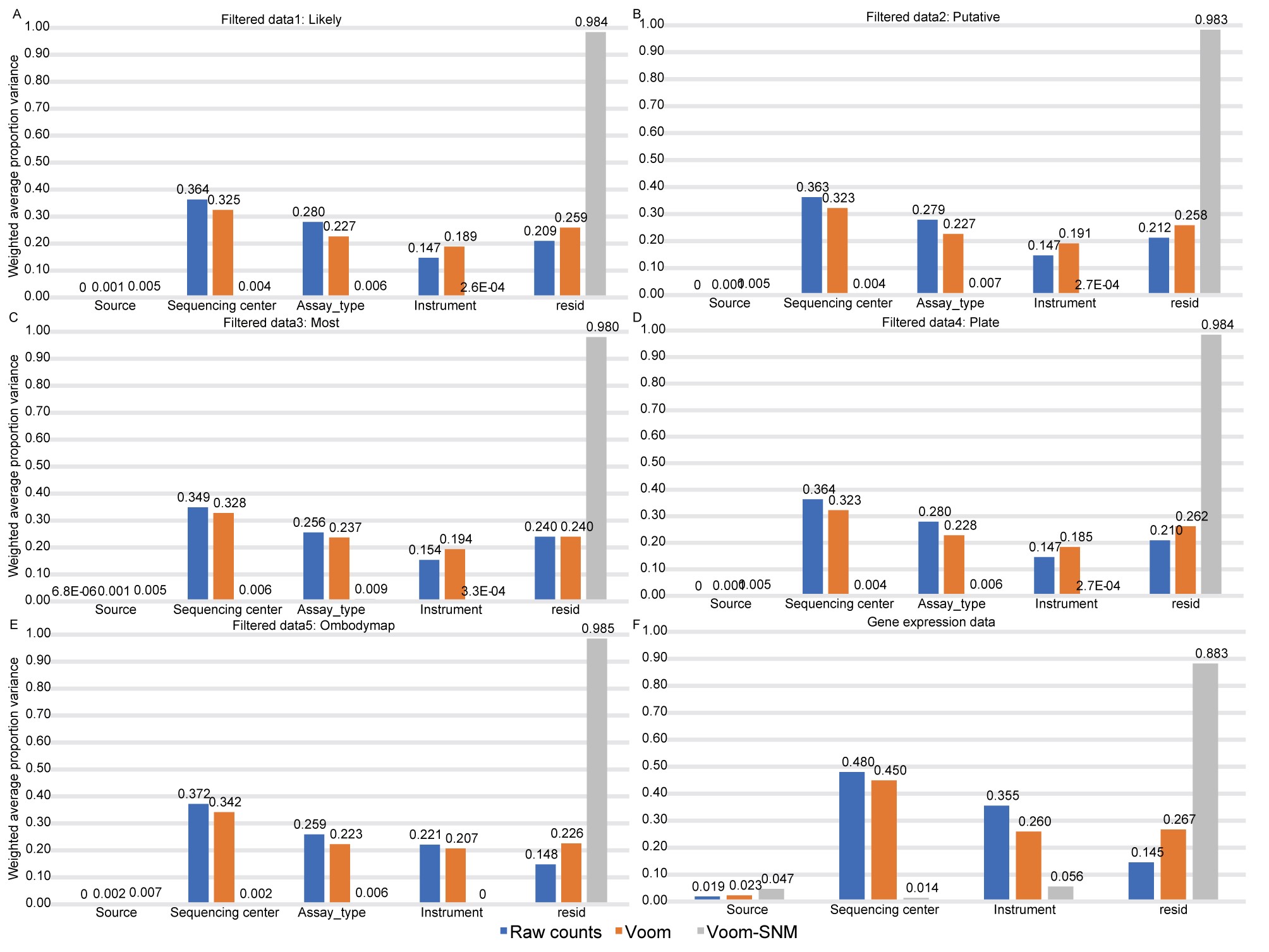

Supplement: FigureS1_bbaf057 [file figures1_bbaf057.jpeg]

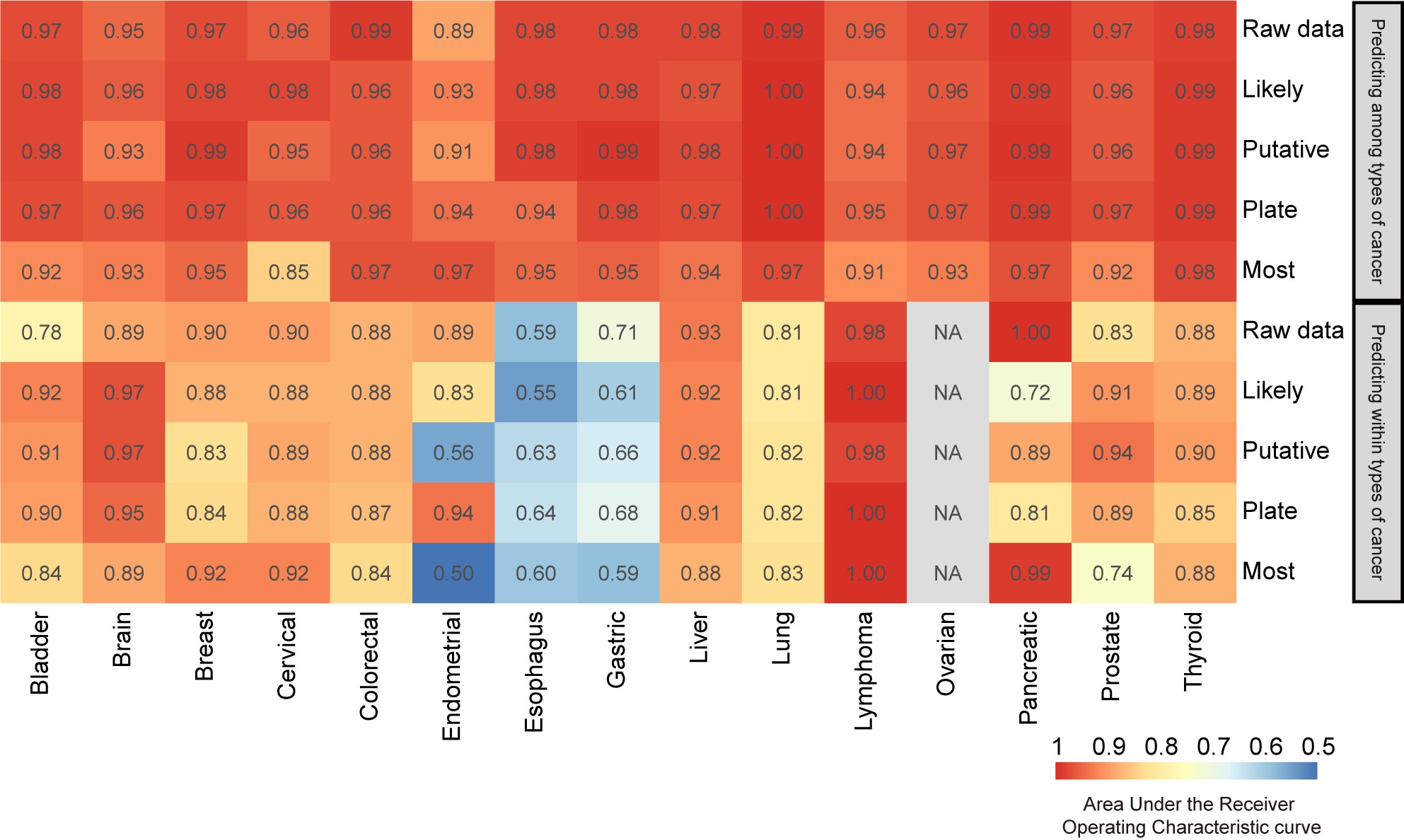

Supplement: FigureS2_bbaf057 [file figures2_bbaf057.jpeg]
